# Decoding Neuropathic Pain: Can We Predict Fluctuations of Propagation Speed in Stimulated Peripheral Nerve?

**DOI:** 10.3389/fncom.2022.899584

**Published:** 2022-07-28

**Authors:** Ekaterina Kutafina, Alina Troglio, Roberto de Col, Rainer Röhrig, Peter Rossmanith, Barbara Namer

**Affiliations:** ^1^Institute of Medical Informatics, Medical Faculty, RWTH Aachen University, Aachen, Germany; ^2^Faculty of Applied Mathematics, AGH University of Science and Technology, Krakow, Poland; ^3^Junior Research Group Neuroscience, Interdisciplinary Center for Clinical Research Within the Faculty of Medicine, RWTH Aachen University, Aachen, Germany; ^4^Institute of Physiology and Pathophysiology, Friedrich-Alexander-Universität Erlangen-Nürnberg, Erlangen, Germany; ^5^Theoretical Computer Science, Department of Computer Science, RWTH Aachen University, Aachen, Germany; ^6^Institute of Physiology, Medical Faculty, RWTH Aachen University, Aachen, Germany

**Keywords:** neuropathy, machine learning, pain, spike count, neural encoding, microneurography, activity-dependent slowing

## Abstract

To understand neural encoding of neuropathic pain, evoked and resting activity of peripheral human C-fibers are studied *via* microneurography experiments. Before different spiking patterns can be analyzed, spike sorting is necessary to distinguish the activity of particular fibers of a recorded bundle. Due to single-electrode measurements and high noise contamination, standard methods based on spike shapes are insufficient and need to be enhanced with additional information. Such information can be derived from the activity-dependent slowing of the fiber propagation speed, which in turn can be assessed by introducing continuous “background” 0.125–0.25 Hz electrical stimulation and recording the corresponding responses from the fibers. Each fiber's speed propagation remains almost constant in the absence of spontaneous firing or additional stimulation. This way, the responses to the “background stimulation” can be sorted by fiber. In this article, we model the changes in the propagation speed resulting from the history of fiber activity with polynomial regression. This is done to assess the feasibility of using the developed models to enhance the spike shape-based sorting. In addition to human microneurography data, we use animal *in-vitro* recordings with a similar stimulation protocol as higher signal-to-noise ratio data example for the models.

## Introduction

Microneurography is an electrophysiological technique of recording peripheral nerve fibers' activity in awake human subjects (Hagbarth and Burke, 1977). It allows the researchers to collect data from C-fibers: unmyelinated thin fibers linked to encoding of different sensory sensations, especially pain, itch, and temperature or serving as efferent nerve fibers for the autonomous nervous system (Ackerley and Watkins, 2018). This way microneurography becomes a unique tool to study the neural signaling of pain and itch in healthy subjects and in patients with different pathologies of the peripheral neural system. There is a major technical problem, which makes this area of research particularly challenging. The signal is recorded extracellularly, so it typically contains overlapping activities of several fibers. There is only little number of available recordings, where the experimenters were able to find a needle placement such that there is a single fiber with very strong signal present and the rest of the fibers could be neglected.

In *in-vitro* recorded data, the fibers can be separated with a certain level of success using tools, such as template matching (Gerstein and Clark, 1964) or clustering (Rey et al., 2015); however, their success is based on the consistency of spike shapes across fibers. Unfortunately, in awake human subjects, the slight patient movements, drift of the microelectrode, changing peripheral activity of sympathetic nerves, and other factors during the recording are causing insufficient reliability of the spike shape alone.

**Figure 1:**
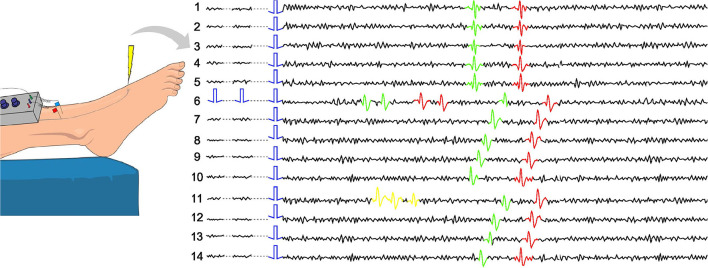
Example of the marking method in two-fiber recording. Each line represents a time interval of the recorded data which is locked to a given background electrical stimulus (single blue square wave). The electrically induced action potentials of two different nerve fibers are shown in green and red with slightly different latency. They can be sorted to the respective fiber by their stable latency (conduction velocity). When two extra electrical pulses are applied (line 6) and the fibers discharge each two action potentials, the response to the regular electrical stimulus is delayed. If there is spontaneous discharge (line 11), it is currently not possible to sort the exact spikes to the respective fiber. It can only be deduced that the fiber with the greater latency shift (green) has fired more action potentials before the latency shift than the other nerve fiber (red).

To deal with this problem, in microneurography, there exists an approach called “marking method” (see Figure 1 and Schmelz et al., 1995 for more details). The basic idea is that during the recording process, *background* electrical stimulation of the nerve ending is applied. Typically, frequencies of 0.25 or 0.125 Hz are used. This stimulation allows the researchers to track the responses of the main fibers involved in the recording, as under similar stimulation, the time between the stimulus and recorded action potential remains close to constant. In Figure 1, the case of two fibers is illustrated. The fibers are slightly different in their propagation speed, which allows to observe their separated responses. Further, using standardized protocols for electrical stimulation (Serra et al., 1999; Weidner et al., 1999) in combination with natural stimuli like mechanical stimulation, it is possible to classify the fibers functionally. The best-known categories are CM (mechano-sensitive C-fiber) or class 1a and CMi (mechano-insensitive C-fiber) or class 1b, which can be further subdivided into more categories.

Low-frequency background stimulation results in similar latencies of the responses, while higher frequencies typically smoothly increase the latencies (Serra et al., 1999). If an additional stimulation is introduced in a form of electrical pulses or mechanical force, we typically observe a sudden latency increase (see line 6 in Figure 1). If there is spontaneous neuropathic firing present (see line 11 in Figure 1), similar latency increases can be observed.

Those latency changes, called activity-dependent slowing (ADS) are directly linked to the changes in fiber speed propagation, as discussed in earlier works on microneurography (Schmelz et al., 1995; Namer et al., 2019), and were studied extensively in the last decades (Schmelz et al., 1995; Bostock et al., 2003; Dickie et al., 2017). The research is linking the number of preceding spikes to latency changes, typically *via* linear approximations. It is also known that the timing of extra stimuli relative to the background pulse and the frequency influences ADS.

We hypothesize, that a reliable model of the dependencies between the preceding activity and resulting ADS can help us to understand the encoding of pain in two ways. First, it will provide us with a better understanding of activity-induced changes in the fiber speed propagation and therefore an understanding of peripheral signal processing which is encoding the stimulus in its quantity and quality (Weidner et al., 2002). Second, it can be used to accept or reject spike sorting decisions based on different spike-shapes features and provide more reliable spiking patterns of single C-fibers, which can be further statistically analyzed to compare evoked and spontaneous firing patterns in different groups of human volunteers.

In this article, we employ simple machine learning algorithms (polynomial regression) to study the dependency between the history of fiber's spiking activity in the past and the resulting latency. The objective behind the choice of this simple modeling approach is that it is easy to understand and interpret. Since the proposed way of accessing the activity history is at the developmental stage, it is important to track the dependencies between different variables.

In previous works, such as (Weidner et al., 2000), the experimental researchers reported a strong influence of the parameters, such as the number of spikes and their distance from the next background stimulus on the subsequent latency. In our preliminary research based on mechanically stimulated rat nerve fiber, we showed the feasibility of capturing this information by dividing the fiber history into intervals of adaptive sizes and building a multivariate regression model of the ADS (Troglio et al., 2021). Here, we continue this work by taking multiple differently stimulated fibers, both animal and humans, and arranging the emerged in Troglio et al. (2021) ideas into a structured approach, so that searchable parameter space can be defined and explored. We use polynomial regression as a model because of its good interpretability. It is important that at this stage of the research we can track the influence of each variable and understand the dependencies between the variables. We start by building individual models for the studied fibers and presenting promising results, as well as encountering challenges. Further, we test the inter-fiber model generalizability and identify the challenges of the available data sets and possible future solutions.

## Methods

### Data

In this project, we used data recorded from isolated mice and rat peripheral nerve fibers and from human peripheral C-fibers recorded with microneurography technique. Both techniques are described in detail previously (Hagbarth, 1979; Weidner et al., 1999; Uebner et al., 2014). There are strong limitations in the choice of data sets which can be used for testing machine learning methods. We are using *in-vitro* recorded animal data, where the noise level is low and the Spike2. template matching for fiber separation can be reliably used in combination with expert knowledge. *In-vivo* human data typically has the above-described problem of multiple fiber recordings, but we can use several exceptionally successful recordings where there is a single prominent fiber, and its action potentials can be labeled by a human expert. In both cases, the data preparation requires a lot of work, and the selection is suboptimal from the point of view of machine learning methods.

**Figure 2:**
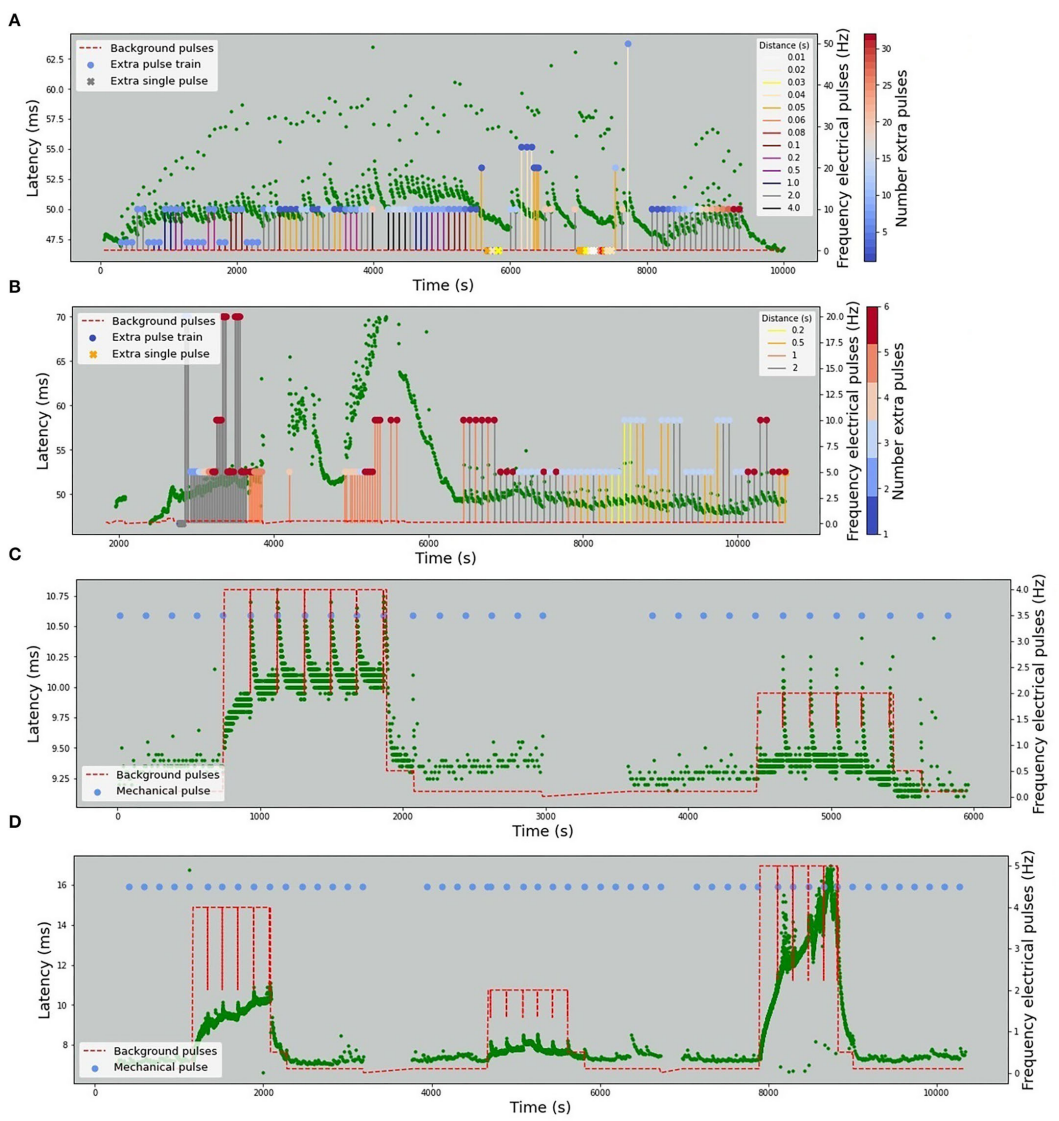
Animal *in-vitro* data. The figures depict four animal experimental protocols. The green dots represent the latencies of responses with x-values corresponding to the onset of the detected spike and y-values corresponding to the response latency in milliseconds. The red dashed line represents the frequency of the background electrical stimulation in hertz. **(A)** MES-I (Mouse Electrical Stimulation, set I). **(B)** MES-II (Mouse Electrical Stimulation, set II). **(C)** RMS-I (Rat Mechanical Stimulation, set I). **(D)** RMS-II (Rat Mechanical Stimulation, set II). In **(A,B)** the colored circles correspond to the number of pulses in an additional electrical stimulation train. In case of a single extra pulse, it is marked as cross. The color of the lines attached to the colored circles depicts the frequency of the extra pulses. In **(C,D)** the blue dots denote the time of mechanical stimuli.

**Figure 3:**
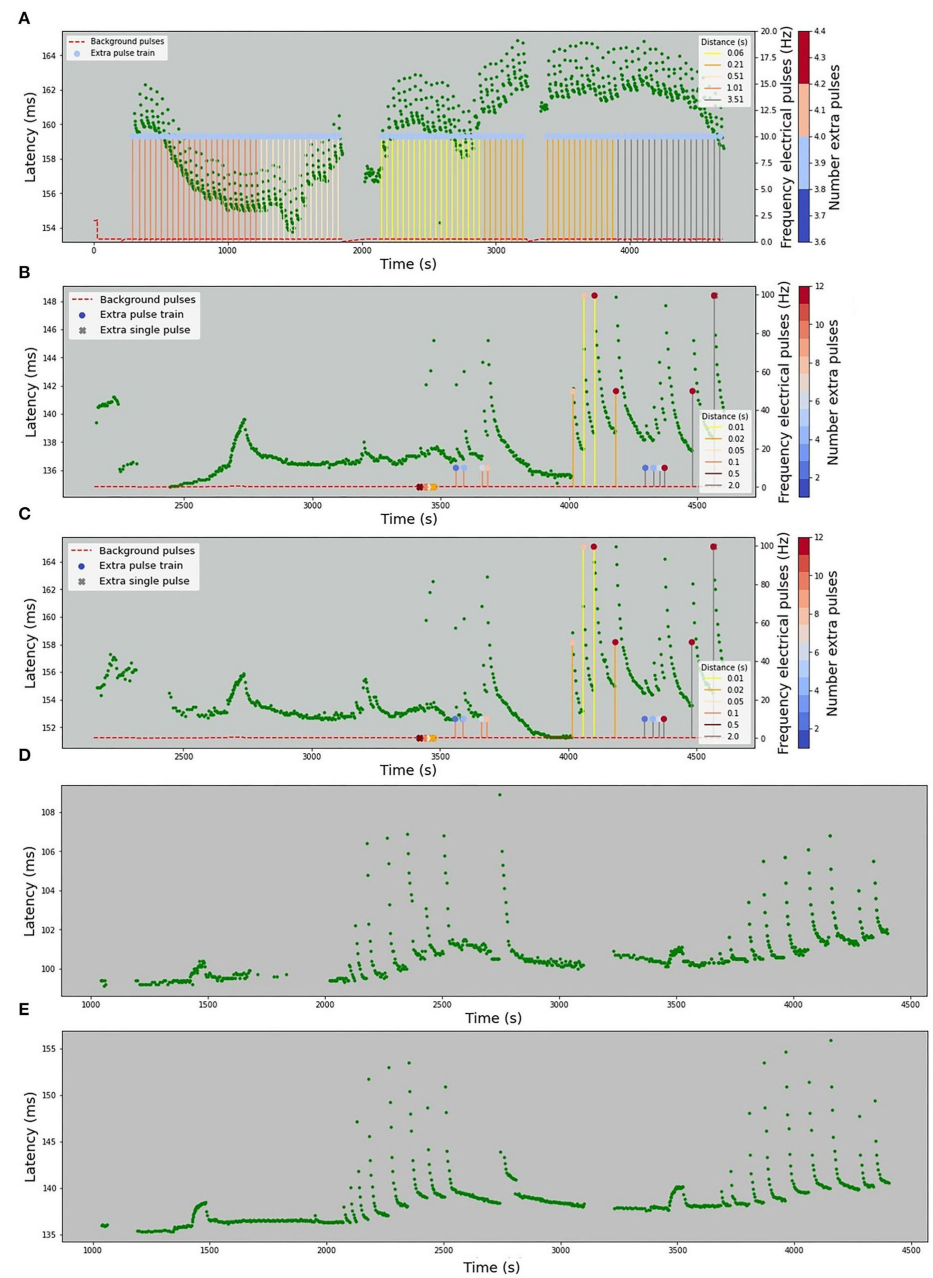
Human microneurography (*in-vivo*) data. The figures depict five human experimental protocols. The green dots represent the latencies of responses, with *x*-value corresponding to the timestamp of the detected spike and *y*-value corresponding to the response latency in milliseconds. Red dashed line represents the frequency of the background electrical stimulation, in hertz. Colored circles correspond to the number of pulses in additional electrical stimulation train. In case there is a single extra pulse, it is marked as cross. The color of the lines attached to the colored circles depicts the frequency of the extra pulses. For patient 2, the complete protocol is not available and only the latencies are plotted. **(A)** HES-I (Human Electrical Stimulation, set I). **(B)** HES-II, P1a (Human Electrical Stimulation, Patient 1, Fiber a). **(C)** HES-II, P1b (Human Electrical Stimulation, Patient 1, Fiber b). **(D)** HES-II, P2a (Human Electrical Stimulation, Patient 2, Fiber a). **(E)** HES-II, P2b (Human Electrical Stimulation, Patient 2, Fiber b).

The stimulation protocols for all datasets are visualized in Figures 2, 3. The y-axis values of the main time series (green dots) correspond to the latencies of the fiber responses to the background stimulus, called further “latencies”. In all data sets, we observe rapid jumps related to additional stimulation, called “extra latencies” as opposed to “main latencies” for intervals with no extra stimulation. After such jumps, we typically observe subsequent latency relaxation to the lower values. Some data sets have visible drifts related to the high frequency of the background stimulation or temperature-related drifts in case of the HES-I set.

#### Mouse Electrical Stimulation Protocol I (MES-I)

The background electrical stimulation is at 0.125 Hz throughout the whole record duration. Additional electrical stimuli are introduced in every 10th regular interval with three varying parameters: number (from 1 to 32), frequency (0 for a single pulse, otherwise 2–50 Hz), and the distance to the next regular stimulus (10–4,000 ms).

#### Mouse Electrical Stimulation Protocol II (MES-II)

This fiber is also only electrically stimulated, but the regular frequency changes. In the first interval (up to 4,000 s), the background stimulation is 0.25 Hz and later changes to 0.125 Hz. The number of pulses of the additional stimulation varies from 1 to 6, the distance to the next regular pulse from 200 to 2,000 ms, and the frequency from 0 (single pulse) to 20 Hz.

#### Rat Mechanical Stimulation Protocol I (RMS-I)

A rat fiber with background pulses at 0.1, 0.5, and 4 Hz. Additionally, mechanical stimulation with a half-sine shape of the applied force is introduced typically every 180 s. The length of the sine wave remains 250 ms and the amplitude varies from 8 to 12 mN. The mechanical stimulus triggers a train of 20–30 action potentials.

#### Rat Mechanical Stimulation Protocol II (RMS-II)

This fiber is similar to RMS-I, though it is most likely a different fiber subtype with much higher reactivity. Background electrical stimulation is applied at 0.1, 0.5, 2, and 5 Hz. The half-sine mechanical pulses last for 250 ms and have amplitudes of 14–15 mN. The mechanical stimulation is typically applied every 180 s with two breaks. A mechanically evoked spike train contains 10–30 action potentials.

#### Human Electrical Stimulation Protocol I (HES-I)

The first human data set with only electrical stimulation has a background stimulation frequency at 0.25 Hz. The frequency of extra pulses is always 10 Hz and 4 pulses with varying distances from 60 to 3,500 ms.

#### Human Electrical Stimulation Protocol II (HES-II: P1a, P1b, P2a, P2b)

The human data sets P1a and P1b are stimulated at 0.25 Hz and for a short period of time at 0.5 Hz (at 2,700 s). There are single extra pulses and multiple extra pulses. The distance to the next regular stimulus ranges from 10 to 2,000 ms and the frequency of extra pulses from 10 to 100 Hz. The number of additional pulses varies from 2 to 12. For the data sets P2a and P2b, the protocol is not available.

### Data Preparation

#### Spike Extraction

**Figure 4:**
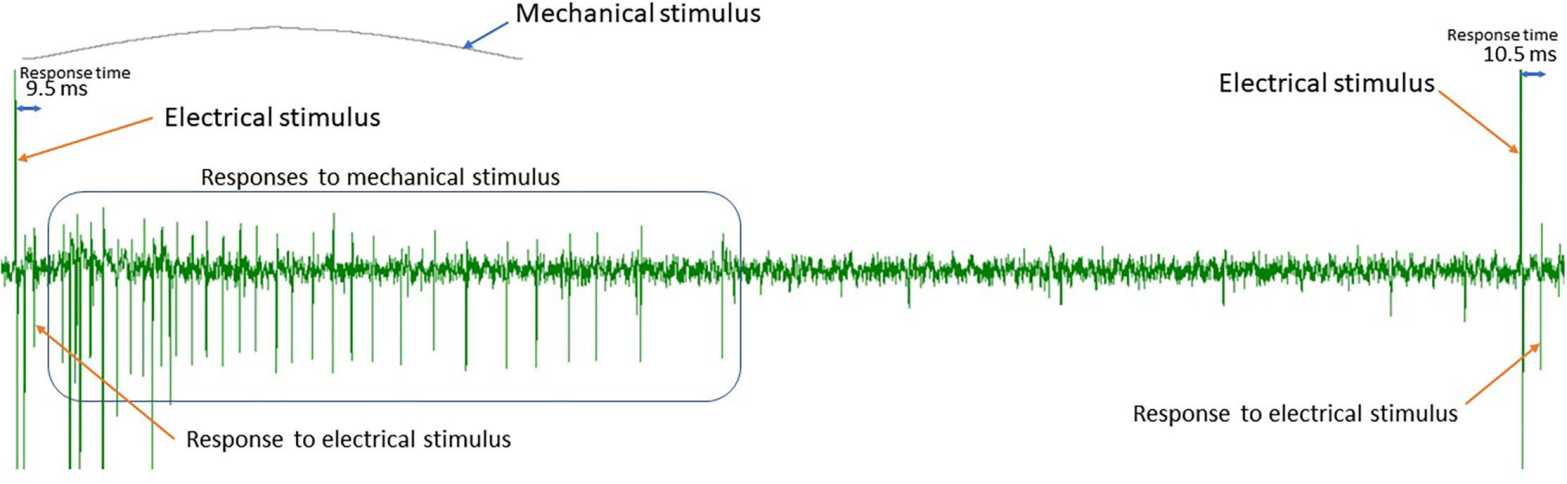
Spike train resulting from mechanical stimulation with a half sine-shaped mechanical force.

There are two types of spikes that we distinguish in this work (see Figure 1). First, the responses to the regular continuous background stimulation (see Section Data), whose latency is dependent on the absence or presence of additional stimuli in the preceding time interval. The second type of spikes is action potentials corresponding to additional stimulation or spontaneous fiber activity. In principle, a healthy fiber should present very limited spontaneous activity, and therefore for additional electrical stimuli, we expect the number of spikes to be approximately equal to the number of stimulation pulses. For mechanical stimulation, the number of spikes depends on multiple factors, such as applied mechanical force or frequency of the background stimulation (see Uebner et al., 2014 for more details). Figure 4 illustrates the half-sine-shaped mechanical stimulation used in the presented protocols and the resulting exemplary spike train.

All animal data and HES-I set are analyzed with Spike2. (2022). The spikes are identified, and shape-based templates are created using expert-defined thresholds. The templates are further merged by the expert *via* visual tracking of the consistent latencies to the background stimuli. In animal *in-vitro* data, the number of fibers is limited, and the data quality is high, which enables reliable sorting. In HES-I, the recording captures a very prominent fiber, which also allows sorting with Spike2.

HES-II data were analyzed with Dapsys (DAPSYS; Turnquist, 2016) by manual tracking of the latencies. The latencies of the responses to background stimulation are computed from the onset of corresponding action potentials and the stimulus.

#### Latencies Normalization

The differences between the measured latencies can be very large. With fiber types and individual differences being a recognized factor, the largest absolute differences can be pinpointed to the varying distances between the stimulating and recording electrodes (from millimeters in *in-vitro* rat recording up to 20–30 cm in adult human microneurography).

There exists the data normalization procedure which is standard in most microneurography laboratories: After a break of 2 min without continuous background stimulation the “unconditioned” latency of the first electrically induced spike is taken as baseline latency and all subsequent latencies are divided by this value. Thus, latency changes are always given as percentage of this baseline latency.

In this work, not all available data had this protocol available. However, since this first unconditioned latency of this protocol is usually identical to the minimal latency, we used the minimal latency value which was visually checked for not being an outlier.

#### Data Filtering

HES-I shows strong drifts of the latency due to the temperature changes (as follows from the experimental notes). Since we do not have any information on the temperature fluctuations, in addition to the raw data, we work with the filtered signal, where a high pass filter of 0.005 Hz is applied. There was a single latency in the filtered data that was an outlier (at 2,500 s), which was removed based on the latency range of the other action potentials.

### Variables of the Models

In the constructed models, we consistently use the normalized latency value as one-dimensional output.

The size of the input vector is a varying number which depends on the length of the fiber spike count history. In the preliminary work (Troglio et al., 2021), we learned that the long-term history (more than 1 min) can still influence the latency, but the closer we are getting to the stimulus of interest, the stronger the influence is. Therefore, it is important to be able to consider both: very short (below 200 ms) and very long-time windows, keeping in mind that large input vectors are not suitable for the models based on small data sets.

**Figure 5:**
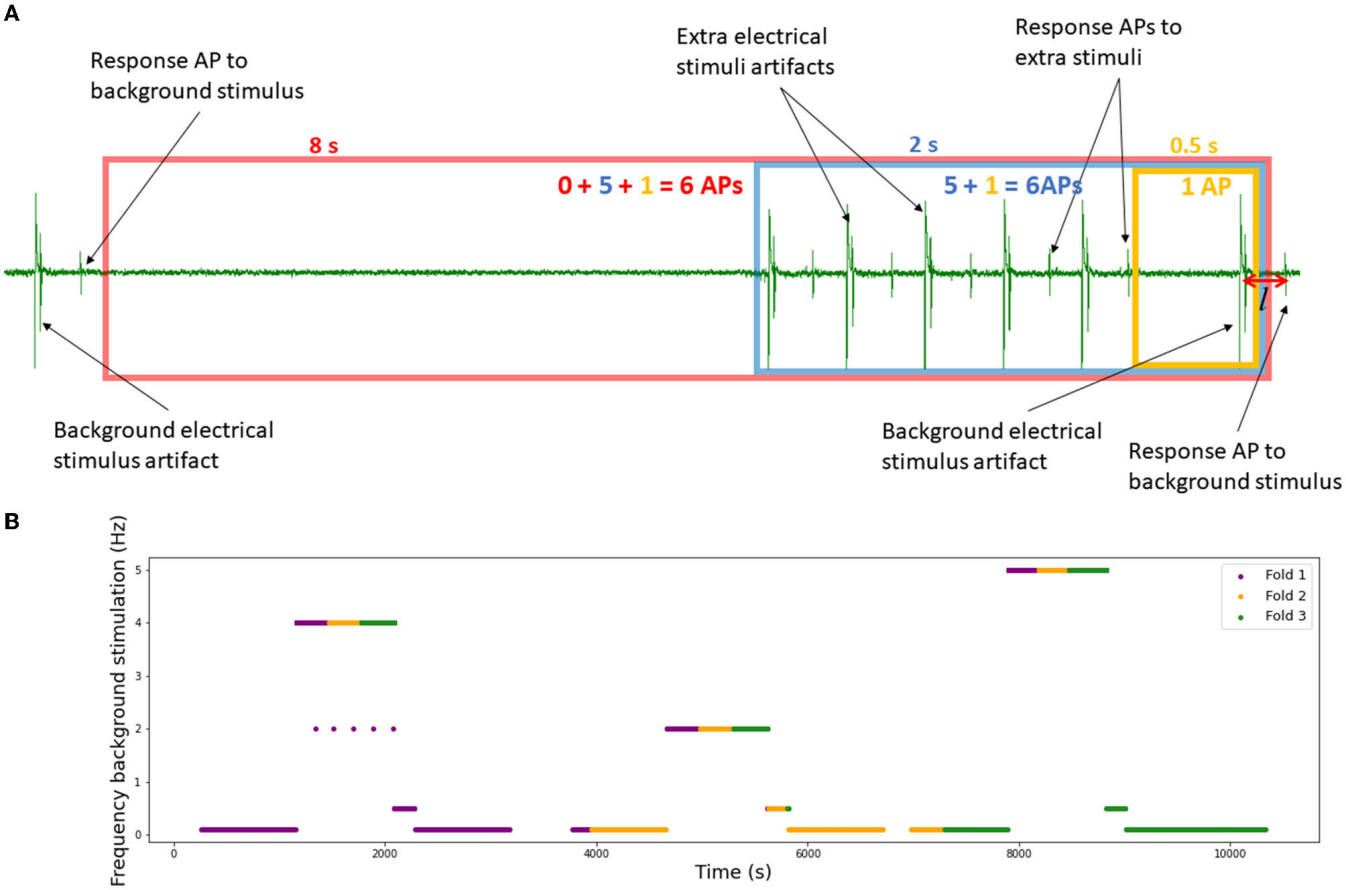
Data preparation. **(A)** Intervals for the spike count. Example with minimal interval length *T*_min_ = 0.5 s and number of intervals *N* = 3. The resulting input vector is (1, 5, 6). **(B)** Background electrical protocol of the set RMS-II which serves as a basis for the 3-fold division. Each different stimulation is divided into three pieces which add up to the final 3-folds (fold 1 in purple, fold 2 in yellow and fold 3 in green).

We propose the following approach to balance the time precision and the size of the feature vector (see Figure 5A).

- For each model, we define the minimal considered history interval (*T*_min_) and the number of intervals *N*.
- For each background stimulus resulting in an action potential with normalized latency *l*, we take *T*_min_ seconds before the stimulus and count the number of spikes within this interval, resulting in number *s*_1_.
- Then, we increase the interval size by a factor of 4, and count *all* spikes there, including those already counted in *s*_1_, resulting in *s*_2_.
- We continue this process until we reach *N* intervals. This way we obtain the input vector [*s*_1_, *s*_2_, ..., *s*_*N*_] and an output value *l*. The largest resulting interval is denoted by *T*_max_.

It is important to note that the time windows overlap and the spikes in smaller intervals are also included in larger intervals (inclusive count). The approach with the exclusive count was tested and rejected, because of its poor performance, possibly due to a large amount of sparse input vectors.

### Predictive Models for Individual Fibers

The investigation was started from building optimized computational models of individual fibers, so that the differences, similarities, and practical problems can be analyzed and compared. Further, we tested the feasibility of model generalizations between the fibers.

#### Train and Test Data Division

Following earlier work (Troglio et al., 2021), we used 3-fold cross-validation. While dividing the data, the following points need to be considered:

- Subsequent latencies are dependent, so the data should be treated as time series and not divided by randomization.
- The densities of latencies are not uniform in time and depend on the protocol. Therefore, we need to use the number of data points instead of the time intervals for the division.
- There are strong drifts in the data which are dependent on the protocol, and therefore different protocols should be represented in all folds.

Thus, in this work, we identified different background electrical protocols and divided each of the identified uniform intervals into three equal pieces, labeled fold 1, 2, and 3. All pieces with the same label form the corresponding fold. Figure 5B shows an illustration of the concept on the RMS-II set as an example. This division does not fully solve the problem of the dependency between data pieces and only the future validation with the leave-one-out approach will answer the question about the realistic model fit.

#### Linear Regression

The basic predictive model used in this article is linear regression, due to its simplicity and interpretability. The degree of the polynomial is a varying parameter denoted as *d*. The implementation of the Python library Scikit-learn (Pedregosa et al., 2011) was used for all computations.

Please note that we tested support vector machines (SVM) and shallow artificial neural networks (ANN) with no essential benefit to the prediction performance. To keep the article structure clear, we only report linear regression model results here. More complex models will be considered again on later stages of the research, when more data becomes available.

#### Filtering Output

After the model is fitted to the train data set, it predicts the latencies for the test set. The predictions are filtered based on the minimum and maximum latency of all latencies. Therefore, predictions that are smaller or larger than the observed latencies are excluded.

#### Parameter Optimization

For each fiber, we perform a separate parameter grid search. The minimal interval length *T*_min_ varies from 0.125 to 1 s; the number of intervals *N* varies from 5 to 10, and the polynomial degree of the regression model *d* varies from 1 to 3.

In this manuscript, we decided not to use a separate data subset for the optimization, neither to use nested folds. The reason is the size of the individual data sets in combination with the focus on investigating of the model feasibility for different types of fibers and stimulations, rather than an interest in exact performance metrics.

The metrics used to decide on the best performance are the *R*^2^-score averaged over all 3-folds. As discussed in Troglio et al. (2021), it remains a suboptimal metric as it is highly prone to large drifts on cost of local dynamics, and we will work on an improved score in future research.

For the best models of each fiber, we track the coefficients of the corresponding regression models, so that we can better understand the influence of each input variable.

### Multi-Fiber Validation

The regression models were tested for inter-fiber generalizability. Most data sets represent different fiber types and different protocols, so the goal was to understand the main issues relevant for the generalizability rather than obtain an optimal model fit.

We checked the performance of the following combinations:

- Best performing model parameters of both sets are trained on RMS-I and tested on RMS-II and vice versa.
- Best performing model trained on MES-I is tested on MES-II and vice versa.
- P1a, P1b, P2a, and P2b sets represent mechano-sensitive C-fibers (CM) from two patients, stimulated with similar electrical protocols. We checked all possible combinations, that is 4 for the same patient data and 8 for two different patients.

We did not use the HES-I set, as the raw data has too strong temperature-related drift and the filtering causes scale mismatches.

Note that in the future, we aim to train the model on a large amount of data collected from a specific uniform group of fibers (e.g., healthy CM) and to be able to predict the latencies for a previously unseen fiber of the same type. In such a setup, leave-one-out validation will become possible.

## Results

### Individual Fibers

**Figure 6:**
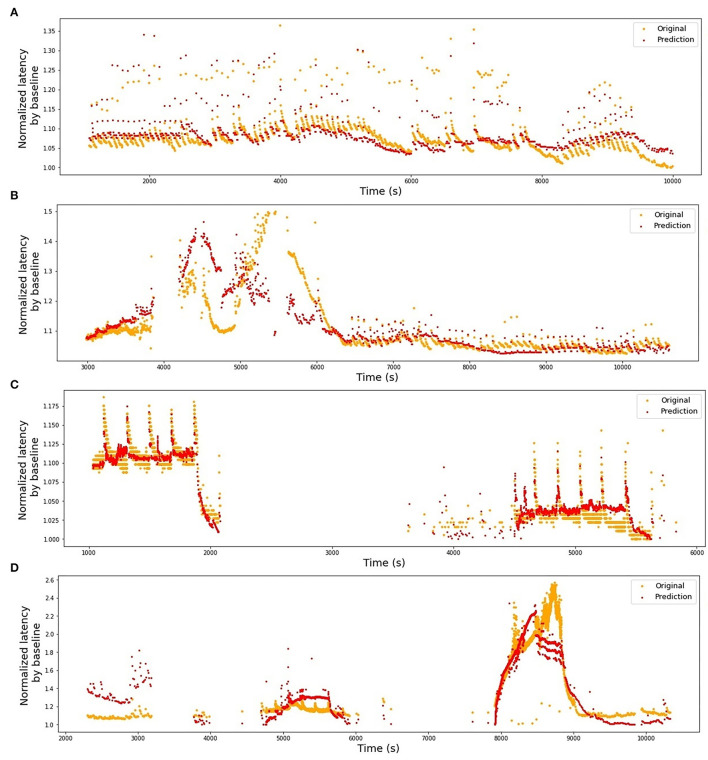
Original latencies from animal fibers and the predicted latencies. **(A)** MES-I, *R*^2^ = 0.64. **(B)** MES-II, *R*^2^ = 0.16. **(C)** RMS-I, *R*^2^ = 0.90. **(D)** RMS-II, *R*^2^ = 0.75.

**Figure 7:**
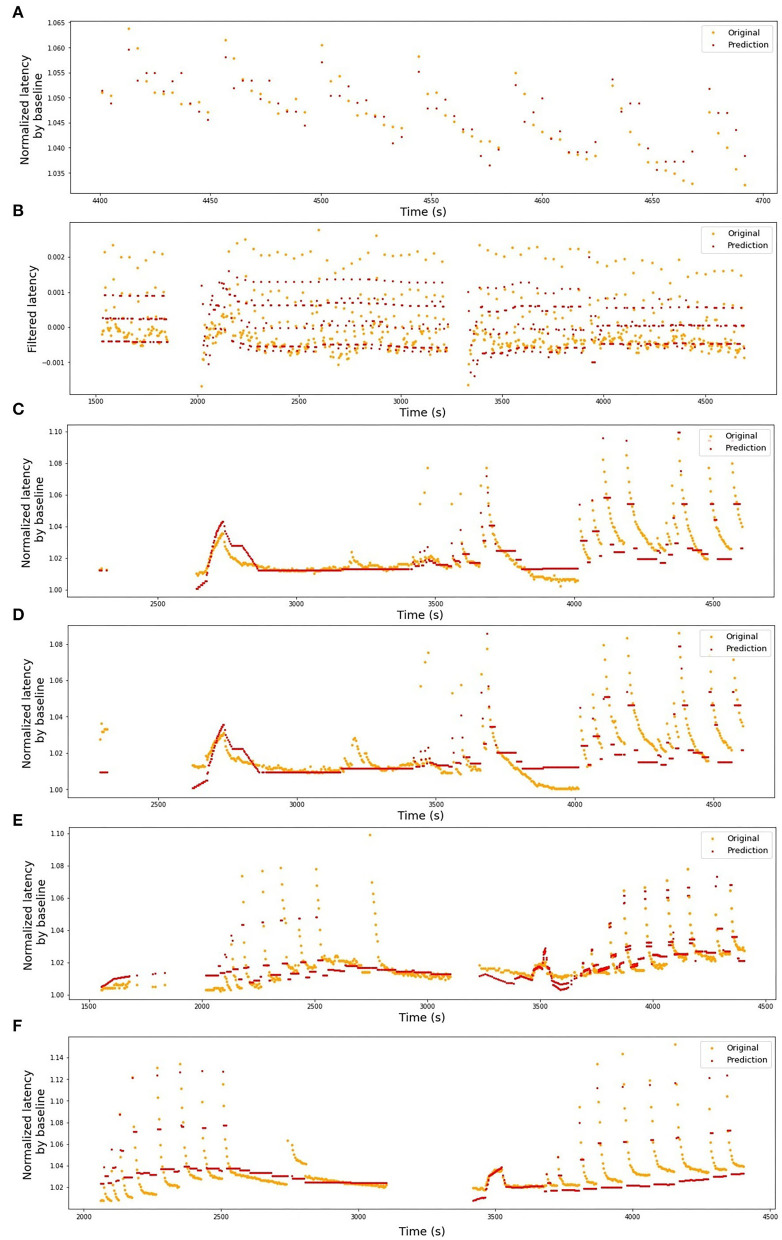
Original latencies from human fibers and the predicted values. **(A)** HES-I, raw data, *R*^2^ = 0.49. **(B)** HES-I, filtered data, *R*^2^ = 0.54. **(C)** HES-II, P1a, *R*^2^ = 0.63. **(D)** HES-II, P1b, *R*^2^ = 0.45. **(E)** HES-II, P2a, *R*^2^ = 0.63. **(F)** HES-II, P2b, *R*^2^ = 0.60.

In Table 1, we present the results of the optimized models for each fiber. The total number of latencies is reported to give information about the size of train and test sets. Parameters *d*, *T*_min_, *T*_max_, and *N* are as defined in the previous section. The last column reports the *R*^2^ averaged over the 3-folds. The graphs presenting the experimental data and the corresponding predicted values can be found in Figure 6 (animal data) and Figure 7 (human data). The prediction is made by training the optimized model on 2-folds and using it to predict the outputs for the third fold. The predicted values for 3-folds are plotted together in the same figure (red). HES-I set is evaluated and plotted both in its raw form and after applying the high pass filter for the drift removal.

**Table 1:** Summarized results of the optimized individual regression models per fiber.

**Fiber**	**Total number of latencies**	***d* (Polynomial degree)**	**T_min_ (Minimal time)**	**T_max_ (Maximal time)**	***N* (Number of intervals)**	** R^2^ averaged over 3 folds**
MES-I	1,197	1	0.250	1,024	7	0.64
MES-II	1,245	1	1.000	1,024	6	**0.16**
RMS-I	6,904	1	0.250	1,024	7	**0.90**
RMS-II	10,560	1	0.500	2,048	7	0.75
HES-I (raw)	961	1	1.000	4,096	7	0.49
HES-I (f)	910	1	1.000	1,024	6	0.54
HES-II P1a	567	2	0.125	128	6	0.63
HES-II P1b	568	2	0.500	128	5	0.45
HES-II P2a	872	1	0.500	512	6	0.63
HES-II P2b	769	1	1.000	1,024	6	0.60

*Total number of latencies reflects the data set size, d denotes the degree of the polynomial model, T_min_ and T_max_ denote correspondingly the minimal and the maximal time interval for the spike count, and N denotes the number of intervals.*

*The bold values show the best and worst R^2^ values of all predictions for each fiber*.

Most fibers showed the superiority of the first-degree polynomials, and Table 2 presents three chosen animal fibers with the coefficient's values corresponding to the spike counts in the listed intervals to illustrate the significance of the features. Since each fold of the 3-fold approach trains a different model, we present all three versions to ensure the robustness of the results.

**Table 2:** Coefficients of the three chosen animal models of degree *d* = 1.

**Fiber**	**Length (in seconds) of time intervals where the spikes are counted**	**Fold 1**	**Fold 2**	**Fold 3**
MES-I	0.25	0.089650	0.076663	0.089589
	**1**	**−0.002876**	**−0.002616**	**−0.004198**
	4	0.009672	0.009656	0.008564
	16	0.005120	0.003784	0.004783
	64	0.000940	0.001006	0.001074
	256	0.000918	0.000891	0.000436
	1,024	0.000378	0.000269	0.000276
	Intercept	0.941203	0.955804	0.984416
RMS-I	**0.25**	**−0.005113**	**−0.004380**	**−0.004438**
	1	0.000759	0.000899	0.000872
	4	0.000651	0.000634	0.000463
	16	0.000679	0.000603	0.000574
	64	0.000070	0.000023	0.000027
	256	0.000068	0.000075	0.000075
	1,024	0.000003	0.000004	0.000004
	Intercept	0.945311	0.952889	0.957989
RMS-II	0.5	0.096538	0.077731	0.088526
	**2**	**−0.006505**	0.001612	**−0.002157**
	8	0.001181	0.003510	0.000047
	32	0.002351	0.002083	0.000314
	128	0.000492	**−0.000003**	0.000472
	512	0.000106	0.000293	0.000213
	2048	0.000184	0.000094	0.000010
	Intercept	0.424364	0.576478	0.905347

*The bold values show the negative coefficients for all models.*

### Inter-fiber Generalizability

**Figure 8:**
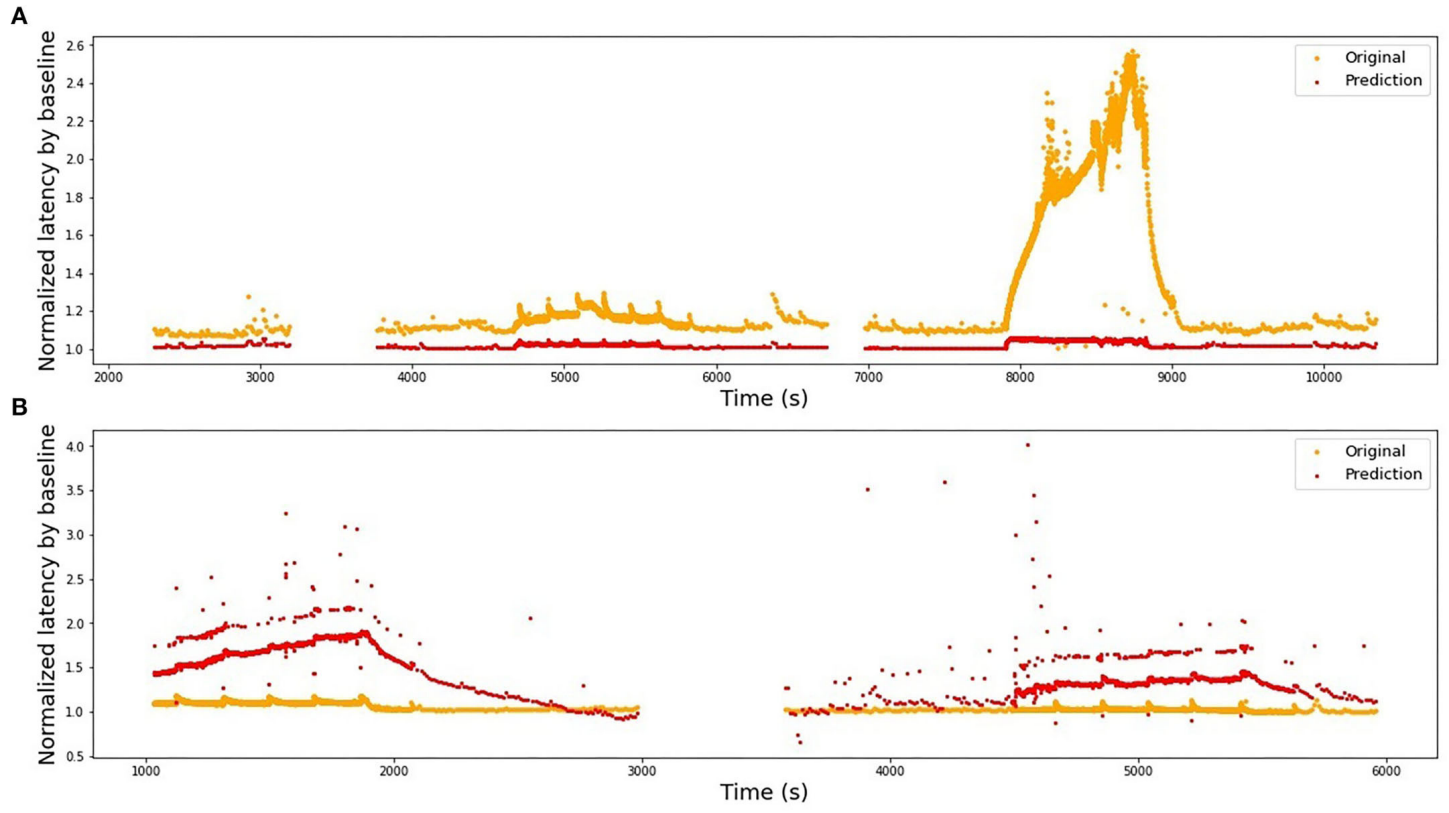
Bidirectional generalizability of the models built on animal fibers with additional mechanical stimulation. The fibers belong most likely to different subtypes, hence the scale of their reaction to the similar stimulation differs strongly and the models cannot be directly generalized. **(A)** Model is trained on RMS-I and tested on RMS-II, R^2^ = −1.62. **(B)** Model is trained on RMS-II and tested on RMS-I, R^2^ = −135.25.

**Figure 9:**
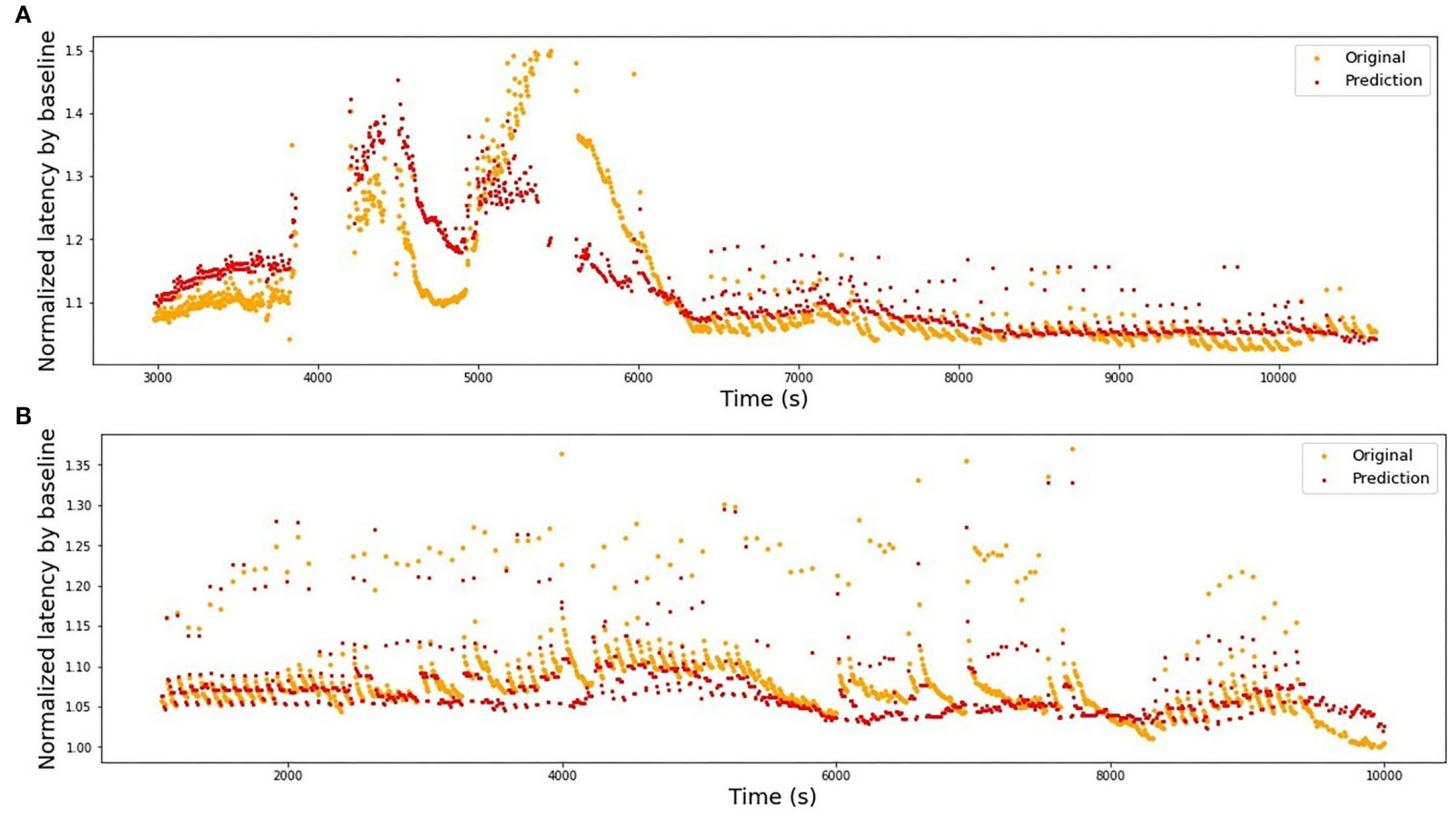
Bidirectional generalizability of the models built on animal fibers with additional electrical stimulation. **(A)** Model is trained on MES-I and tested on MES-II, R^2^ = 0.55. **(B)** Model is trained on MES-II and tested on MES-I, R^2^ = 0.60.

We use the best-performing model for a single fiber as a train set (see Table 2), train it on the complete fiber instead of 2-folds and then compute the *R*^2^-score for the test fiber. The results of bidirectional generalizability tests of the animal data with the mechanical and extra electrical stimulations are presented in Figures 8, 9. The *R*^2^-scores are negative for the mechanical data as the two sets have completely different scales of latency values. The scores for the fibers MES-I and MES-II (0.55 and 0.45) support the feasibility of the generalization.

**Figure 10:**
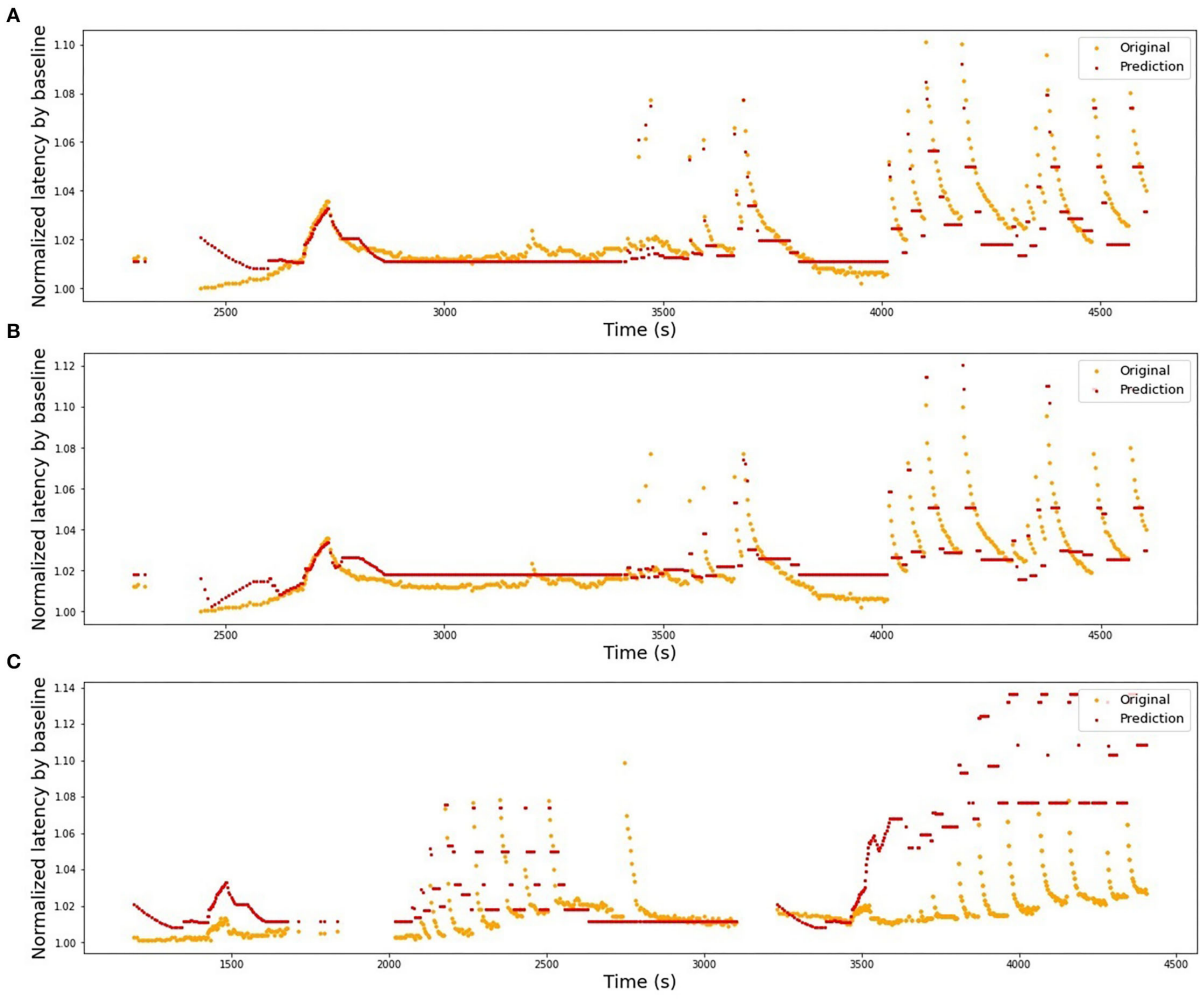
Selection of illustrations of generalizability performance in the HES-II set. **(A)** Model is trained on P1b and tested on P1a (same patient, best performing pair of fibers), *R*^2^ = 0.84. **(B)** Model is trained on P2b and tested on P1a (different patients, best performing pair of fibers), *R*^2^ = 0.72. **(C)** Model is trained on P1b and tested on P2a (worst performing combination overall), *R*^2^ = −11.08.

Finally, different combinations of the fibers from HES-II were examined. The full results are presented in Table 3. First, we checked the performance of the model train and tested on different fibers of the same patient (4 combinations) and then the eight combinations of different patients are listed. Figure 10 presents the best results for the same patient fibers and for different patients. Additionally, the worst-performing combination is presented for comparison.

**Table 3:** R^2^-scores for different two-fiber combinations of human fiber models from HES-II.

	**Train**	**Test**	**R^2^, model optimized on train set**	**R^2^, model optimized on test set**
Same subject	P1a	P1b	0.77	0.77
	P1b	P1a	**0.84**	**0.84**
	P2a	P2b	0.04	−0.20
	P2b	P2a	−5.75	**–**6.07
Different subjects	P1a	P2a	−9.94	**–**8.24
	P1a	P2b	**0.60**	0.01
	P1b	P2a	−11.08	**–**5.14
	P1b	P2b	0.55	**–**0.18
	P2a	P1a	0.06	0.18
	P2a	P1b	0.25	0.31
	P2b	P1a	0.58	**0.72**
	P2b	P1b	0.31	0.55

*The bold values show the best R^2^ values for all two-fiber combinations*.

## Discussion

In the presented project, we studied the feasibility of predicting activity-related changes of conduction velocity in the neural fiber propagation speed. Since changes in conduction velocity, seen as changes in response latency, are dependent on the preceding activity and correlate to its magnitude, they represent the short-term (range of minutes) memory of the nerve fiber and are used as a tool to quantitatively estimate the preceding activity of the nerve fiber in microneurography. Thus, predictive models of latency changes could provide useful information to support spike sorting algorithms, which are based on spike shapes. The combination of latency changes predicting the preceding activity with shape-based spike sorting might increase substantially the accuracy of sorting algorithms for microneurography data with low signal-to-noise ratio, overlaying signals from other nerve fibers and similar spike shapes of different nerve fibers.

Below we will discuss what we learned from the individual fiber models and from the attempts to generalize the models to different fibers. Finally, we will propose the next steps necessary to use this knowledge in practice for the spike sorting problem as it is manifested for the special case of microneurography experiments.

### Individual Fibers

#### Feasibility of the Modeling Approach

The models built on individual fibers showed promising performance quality. Importantly, the proposed approach to the spike counting in time windows increased by a factor of 4 proves to be universal for the very different stimulation protocols. In future work, the adjustment of the interval definition to the specific protocols could be considered. This should be done based on stimulation protocols, since the distance between the additional stimulation and the subsequent background pulse is essential for the latency formation due to the existence of two different molecular processes underlying the changes in response latency (Bucher and Goaillard, 2011). In short-term effects (in a range of <1 s), passive membrane changes as seen in the recovery cycle are properly involved (Weidner et al., 2002; Bostock et al., 2003), whereas long-term effects (range longer than 1 s up to several minutes) might be based on the ion accumulation within the nerve fiber or longer-lasting molecular effects, such as slow inactivation of sodium channels (De Col et al., 2008). Therefore, the interval definition should be directly linked to this distance for improved temporal resolution. The choice of folds is already made dependent on the stimulation protocol, which is important to handle large drifts related to the background stimulation frequency. R^2^ was already reported in Troglio et al. (2021) as a suboptimal goodness-of-fit measure, as it is highly biased by the large drifts of the data set. Drifts can be based on temperature changes of the skin. Especially in in-vivo human recordings, the activity of the sympathetic nervous system due to a “fight or flight” response results in vasoconstriction with concomitant temperature drop. Also, the long-term activity of the nerve fiber can lead to an accumulation of activity-dependent slowing of conduction velocity which is associated with reduced excitability (De Col et al., 2012). For the successful use of the developed models to the spike sorting task, it is important to correctly predict the drifts, but also individual activity-related latency jumps. Therefore, as a first step, we are working on the development of a weighted score, which considers both the large-scale behavior (drift), as well as the small-scale important phenomena, like the fit of the evoked latency jumps and the subsequent recovery to lower values.

In the presented results, we do not observe large differences of the model qualities between in-vitro and in-vivo data. However, it should be noticed that on the preparation level, the proportion of the potentially usable data sets for such modeling is much higher in the collected in-vitro data. Human in-vivo data represents on average much lower signal-to-noise levels, shorter recording times (a challenge for single-fiber modeling) and, most importantly, the issue of fiber separation. In further research, we hope to use latency models built on combined in-vitro and high-quality in-vivo data to support the fiber separation in other human data sets. However, this approach is not feasible for the case of high-level neuropathic firing, as spiking activity may vary strongly and have unpredictable effects on the latency.

#### Common Trends in Individual Fiber Models

All models show similar overall behavior: the automatically optimized length of the largest considered time interval (*T*_max_) is rather large, starting from 128 s for the shortest time series and increasing up to 4,096 s for the HES-I unfiltered data set. This last value of the time window will be limited in future algorithms, preventing the grid search to take *T*_max_ above a certain proportion of the overall record duration. Nevertheless, we can see in Figure 2, particularly in panel (D) (RMS-II), that the switch to higher baseline stimulation (4 Hz) causes a clear drift to higher latencies and as long as the latencies keep growing, the moment of the stimulation change matters and so does the history.

It is likely that the unusually high *T*_max_ in HES-I can be attributed to the model's attempt of translating the temperature-related drift into something explainable in terms of the available input features.

Further, for most fibers, *d* = 1 (linear model) seems to work best. The cause can be the proportion of feature vector size to the available training data points. We expect that when we have larger uniform data sets available, higher degree polynomials will take over the optimization process. Larger data sets will also allow to use full potential of more complex machine learning models.

We tracked the regression coefficients of three chosen models in Table 2. The general tendency is that the further we move from the latency in question, the less weight this history has in the model. It agrees with the results from Weidner et al. (2002). Small coefficients associated with long history intervals suggest that we should consider imposing a limit of maximal history *T*_max_, due to little informative value of the very long-time intervals.

The negative coefficients associated with the interval of 1 and 0.25 s in MES-I and RES-I, could be possibly related to supernormality effect (Weidner et al., 2000; Bostock et al., 2003). In RMS-II, the intervals with negative coefficients are too large for that association. However, the larger intervals are also including smaller intervals in their count (inclusive count), which makes the interpretation difficult. In the current article, the exclusive count was not reported as performing worse than the inclusive count, but we are working on improving this approach by filtering off the sparse features generated by exclusive count.

### Inter-fiber Model Generalizability

Since the fibers are not uniform regarding their sub-types and protocols, there were limited options for the testing model generalizability. We started by studying the bidirectional model generalizability between two pairs of rat and mice fibers: electrically and mechanically stimulated.

Train on MES-I and test on MES-II worked very well, achieving the score much better than the 3-folds average on MES-II alone (*R*^2^ of 0.55 vs. 0.16). The real improvement, as assessed visually by comparison of the Figures 6B, 9A is less impressive and is partially due to the focus of *R*^2^ scores on large data drifts. Nevertheless, the result is still promising.

The second pair is far less successful, which was to be expected looking at the normalized latency values at the analogous parts of the stimulation protocol. RMS-II shows much higher sensitivity levels. In this experiment, it was difficult to control for the isolation of the specific fiber type and there is a high probability, that RMS-I and RMS-II belong to different types, hence such large differences in scales and they are reflected in the feature coefficients as well (see Table 2).

**Figure 11:**
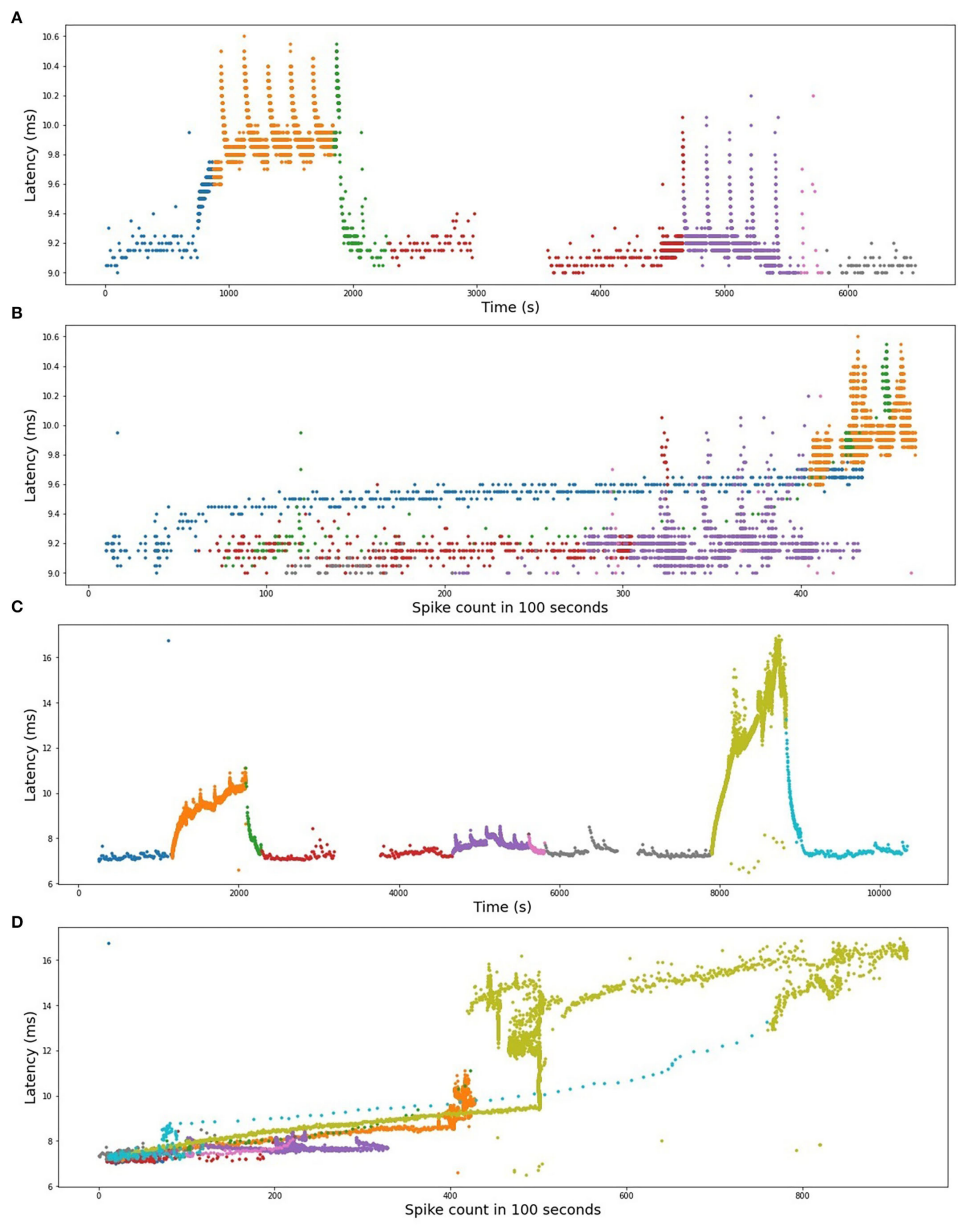
**(A)** RMS-I, time series plot. **(B)** RMS-I, latency plotted as a function of spike count. **(C)** RMS-II, time series plot. **(D)** RMS-II, latency plotted as a function of spike count. In **(A,C)**, the colors represent different frequencies of background protocols. In **(B)** and **(D)**, the spike count is computed in the preceding 100 s.

To illustrate the qualitative similarities between RMS-I and RMS-II, in Figure 11, we present the plots of the latency dependency of the spike count in the preceding 100 s for both fibers. In the time-series plots, the color is varying in accordance with the baseline electrical protocol. The same color is kept in the plots of the latency dependency from the count. In both fibers, we can clearly distinguish linear parts corresponding to the accumulative latency growth right after each increase in the frequency of the baseline electrical protocol. The linear increase of latency being dependent on low-frequency stimulation points to slow axonal accumulating processes. These are not saturating in the range which we observe, but might saturate out of our observation range with longer stimulation periods (De Col et al., 2008). This fits well to the experimental observation of sodium accumulation within the axon with long-term activity (De Col et al., 2008). Less organized jumps are related to mechanically stimulated parts. The decrease of the latency has a rather nonlinear shape, which suggests that there are different molecular processes compared to the processes underlying frequency-dependent latency increase. The latency decrease might be based on a combination of increased sodium/potassium pump activity which is not linearly combined with other processes. We conclude that with a sufficient amount of data higher level polynomials and other nonlinear models need to be used.

In the next step, we studied all pair-wise combinations of four fibers from the HES-II set. The huge advantages of HES-II data are uniform fiber types (CM, mechanosensitive C-fiber), good data quality, similar protocols, and shared subject for P1a/P1b and P2a/P2b, respectively. The within-subject transferability works very well for the first patient, with the models achieving higher scores than on respective single fibers. That can be the result of the increased training data set. It is also important to realize that the two data sets are not independent and therefore the results are not fully reflecting the goodness of the model generalizability.

**Figure 12:**
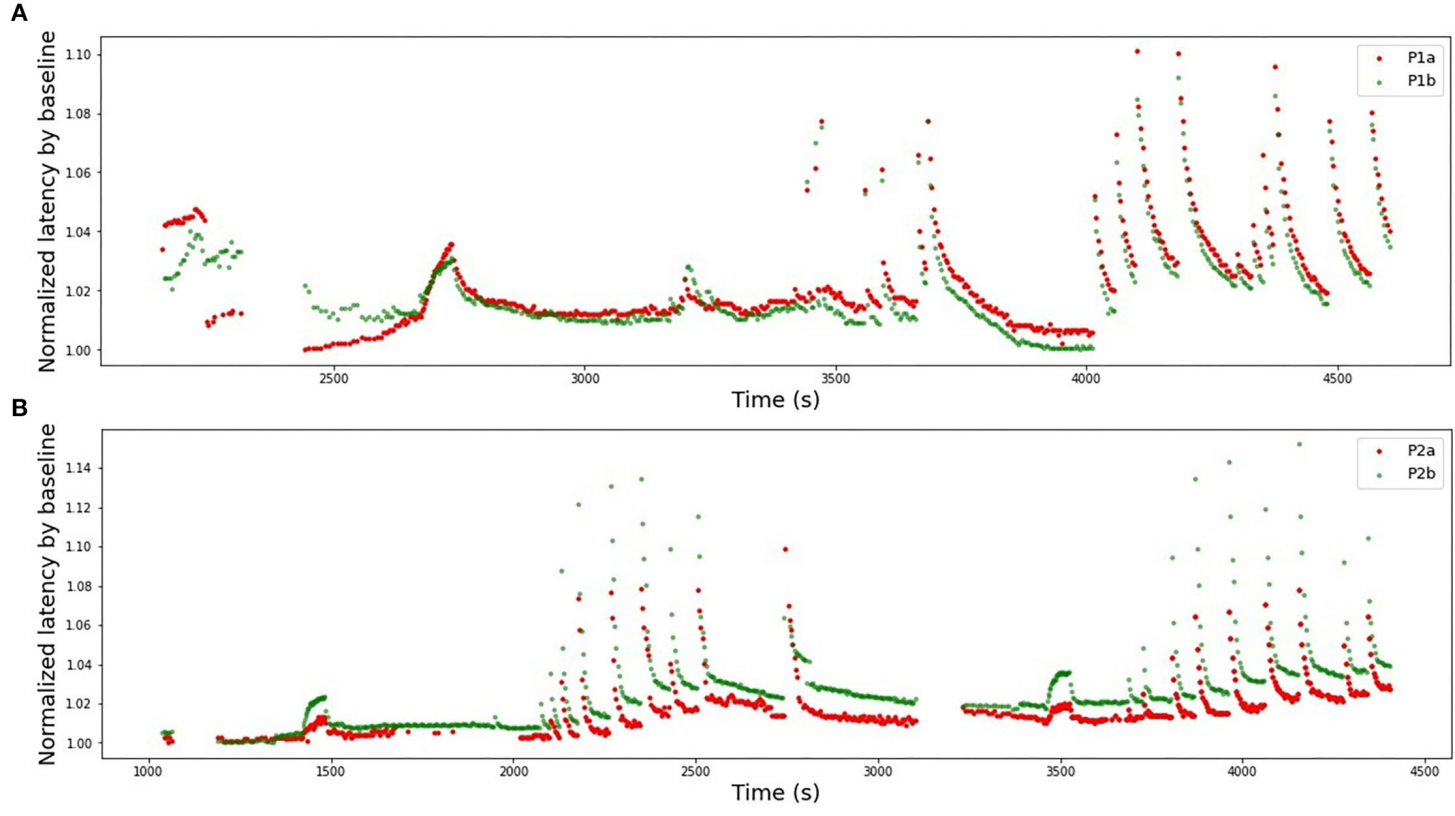
Latencies from two fiber pairs of two different patients (set HES-II). **(A)** Patient 1, fibers P1a, and P1b. Example of successful normalization. **(B)** Patient 2, fibers P2a, and P2b. The normalization is not optimal and the differences between two fibers seem to drift in time, resulting in poor generalizability between the models.

The two models of the second subject show an opposite situation with large negative *R*^2^-scores. This is not a result of bad data quality, as both single fibers showed good results. Rather, the low scores should be attributed to the normalization problem (see Figure 12). In the case of the second subject, our applied normalization was not successful. There seems to be a drift between the two data sets and the distance increases with time. This is explained by the variance in the dependency of latency changes from previous activity in individual nerve fibers which is seen already at the 2 Hz stimulation protocol in Figure 12 before second 1,500 in a different amount of activity-dependent conduction velocity slowing and reduced normalization of the latency of P2b. Since, as discussed above, there are slow processes leading to an accumulation of latency increase and thus the latencies drift apart over time with accumulating preceding activity. The same issue propagated to the inter-subject generalization attempts, with all combinations of P1a, P1b, and P2b much more successful than any model involving P2a.

Interestingly, the performance of models trained on P1a and P1b data and tested on P2b is slightly superior to the opposite direction. If we additionally consider the scores achieved for the parameters optimized for the test set, we can speculate that quadratic terms with enough training data perform better than purely linear combinations.

The large disadvantage of the fitted models (propagating for the single-fiber model) is that the predicted latencies form “steps” instead of attempting to fit the latency recoveries after additional stimulations are applied. This may be possible to fix by improving the goodness-of-fit measurement and giving more weight to the recovery shapes.

### Further Steps

#### Data Quality Improvement and Control

During our study, we identified several major challenges in building successful multi-fiber models which can help us to better understand the pain encoding mechanisms. Firstly, we need to build a database with structurally stored metadata and easy-to-use experimental data. The first steps are already made through developing the data handling platform openMNGlab (Schlebusch et al., 2021), which is using the Neo tool for electrophysiological data handling (Garcia et al., 2014). We are also working on introducing a uniform metadata structure and making it searchable with odML tables (Zehl et al., 2016). This approach will allow us to organize larger data amounts and group it to uniform collections where generalizability is feasible.

Further, due to the experimental protocols, the spiking activity is overrepresented in some parts of the baseline intervals on cost of others. For instance, mechanical stimulation linked to high spike count is always applied very close to the baseline electrical stimulus resulting in high counts overrepresented at this specific position. To support activity diversity, we are working on alternative protocols which can improve model training.

Problems caused by untracked temperature changes are represented by the set HES-I. In future experiments, wherever possible the temperature will be recorded. To handle already available data, we will work on the improvement of the filtering methods.

In our future work, it will be important to introduce standardized data quality assessment and rejection criteria for the full data sets or selected fragments. It can be done in analogy to artifact removal routinely performed in EEG research (Delorme and Makeig, 2004) and incorporated in the openMNGlab pipeline.

#### Methodology Improvement

The normalization process improvement is necessary to handle the problems, such as reported above on HES-II, when P2a and P2b were not well aligned despite the used normalization. There is considerable variance in the dependency of latency changes and preceding activity not only between different nerve fiber classes, but also within a nerve fiber subclass for individual nerve fibers. We may consider repeating a fixed electrical stimulation routine several times and normalize the data piecewise to correct the fiber individuality. An exemplary protocol consisting of 2 Hz stimulation for a certain period (Serra et al., 1999) was used in HES-II data and for the second patient is manifested by two data “hills” at c.a. 1,500 and 3,500 s (see Figures 3D, E). The distance between P2a and P2b increased between those timestamps.

There is an improvement potential in the spike counting interval definition adjusted better to the specific protocol. Particularly, setting the minimal considered time interval *T*_min_ individually can be very helpful. Further, the rejection of the sparse features to avoid unnecessary lengthy input vectors will be considered.

When the amount of readily available uniform data is large enough, it is important to consider more complex models. There has been some preliminary work done toward employing (N)ARX models—autoregressive models with exogenous input (Astrom, 1970; Siegelmann et al., 1997). Supplementary Figures S1, S2 illustrate the results of the ARX implementation with the history of three steps and the spike count in the past 8.1 s as an exogenous input for MES-I and MES-II. Those models showed large potential in handling the issues of drift and normalization.

Importantly, a large analysis-ready data set in combination with a multi-fiber model, where in particular the normalization issues are solved, will allow the creation of a separate set for parameter optimization and the leave-one-out validation approach. This setup will allow us to update the current suboptimal measures of the model fit and obtain more reliable knowledge on the perspectives of fiber activity-to-latency modeling.

Despite the problems, current results can be already tested for practical applications, with one of the most important being spike sorting. We are currently working on preparation of the golden standard data and testing the performance of shape-based spike sorting for the human microneurography data. In the next step, we will test if tracking fiber latency changes can be practically beneficial for the sorting process, particularly if combined with recently developed algorithms that support the handling of data drifts and experimental protocol changes (Davey et al., 2020; Buccino et al., 2022).

## Conclusions

This study is a first step in the direction of using machine learning to create hypotheses about fiber latency changes as readout for molecular mechanisms. We continued the research reported in Troglio et al. (2021) and show for the first time that it is possible to prepare data from microneurographic peripheral nerve fiber recordings for the use of machine learning algorithms and examine the dependency of conduction velocity (latency) from the preceding activity which represents the short-term memory of an axon.

We proposed a systematic approach to the data preparation, feature extraction with balancing feature vector size and precision of the spike location and parameter optimization. The results show that we can predict the fiber latency changes based on the preceding activity even with simple polynomial regression models.

We conclude further that in uniform data subsets with a matching experimental protocol and fiber types, we can expect that the built models can be generalized to previously unseen data. Thus, we might be able to automatically predict (backcast) preceding activity from resulting latency changes, which we can assess *via* conventional analyzing methods in microneurography.

It is particularly relevant in multifiber recordings with low signal-to-noise ratio in which we can only analyze data using the “marking method” and estimate previous activity very roughly *via* visually tracked latency changes. The machine learning approach is expected to support automatic spike sorting algorithms that in turn will allow us to improve our understanding of the peripheral pain signaling mechanisms.

## Data Availability Statement

The datasets presented in this article are not readily available because of ethical and privacy restrictions. Requests to access the datasets should be directed to BN, bnamer@ukaachen.de.

## Ethics Statement

The studies involving human participants were reviewed and approved by Ethics Board of the University Hospital RWTH Aachen, Vo-Nr. EK141-19. The participants provided their written informed consent, and the study was conducted according to the Declaration of Helsinki. The animal study is on animal tissue (*in-vitro*), not subjects. Handling and treatment of animals used for this study was approved by the Responsible Ethics Administration at the Government of Lower Franconia. The use of animals for this study was specifically reported to and approved by the Responsible Ethics Administration of the FAU Erlangen-Nürnberg.

## Author Contributions

The idea was developed by BN and EK. The study was designed by EK, BN, and RR. The experiments and the data collection were conducted by BN and RC. The data were analyzed by AT, EK, and PR. The data were interpreted by BN, EK, AT, RC, RR, and PR. The manuscript was drafted by EK, AT, and BN. The manuscript was revised by all authors. All authors agree to be accountable for the content of the work, contributed to the article, and approved the submitted version.

## Funding

This work was partially funded by the Excellence Initiative of the German Federal and State Governments G:(DE-82) EXS-SF-SFDdM013 and also supported by the IZKF TN1-6/IA 532006. BN was supported by a grant from the Interdisciplinary Centre for Clinical Research within the Faculty of Medicine at the RWTH Aachen University and the German Research Council DFG NA 970 3-1, DFG FOR 2690. EK acknowledges Faculty of Applied Mathematics AGH UST statutory tasks within subsidy of Polish Ministry of Science and Higher Education.

## Conflict of Interest

The authors declare that the research was conducted in the absence of any commercial or financial relationships that could be construed as a potential conflict of interest.

## Publisher's Note

All claims expressed in this article are solely those of the authors and do not necessarily represent those of their affiliated organizations, or those of the publisher, the editors and the reviewers. Any product that may be evaluated in this article, or claim that may be made by its manufacturer, is not guaranteed or endorsed by the publisher.
