# Corrigendum: Decoding neuropathic pain: Can we predict fluctuations of propagation speed in stimulated peripheral nerve?

**DOI:** 10.3389/fncom.2023.1149849

**Published:** 2023-02-06

**Authors:** Ekaterina Kutafina, Alina Troglio, Roberto de Col, Rainer Röhrig, Peter Rossmanith, Barbara Namer

**Affiliations:** ^1^Institute of Medical Informatics, Medical Faculty, RWTH Aachen University, Aachen, Germany; ^2^Faculty of Applied Mathematics, AGH University of Science and Technology, Krakow, Poland; ^3^Junior Research Group Neuroscience, Interdisciplinary Center for Clinical Research Within the Faculty of Medicine, RWTH Aachen University, Aachen, Germany; ^4^Institute of Physiology and Pathophysiology, Friedrich-Alexander-Universität Erlangen-Nürnberg, Erlangen, Germany; ^5^Theoretical Computer Science, Department of Computer Science, RWTH Aachen University, Aachen, Germany; ^6^Institute of Physiology, Medical Faculty, RWTH Aachen University, Aachen, Germany

**Keywords:** neuropathy, machine learning, pain, spike count, neural encoding, microneurography, activity-dependent slowing

In the published article, there was an error in the funding statement. The grant number for the Excellence Initiative of the German Federal and State Governments was listed as “G:(DE-82) EXSSF-SFSynt001” but should be “G:(DE-82) EXS-SF-SFDdM013”. The correct Funding statement appears below.

