# Supplementary material for: Decoding Neuropathic Pain: Can We Predict Fluctuations of Propagation Speed in Stimulated Peripheral Nerve?
Source: Front Comput Neurosci. 2022 Jul 28;16:899584. doi: 10.3389/fncom.2022.899584 (PMC9366140; doi:10.3389/fncom.2022.899584)
Supplement: Supplementary file 1 [file Data_Sheet_1.docx]

Supplementary Material

## Supplementary Figures

**
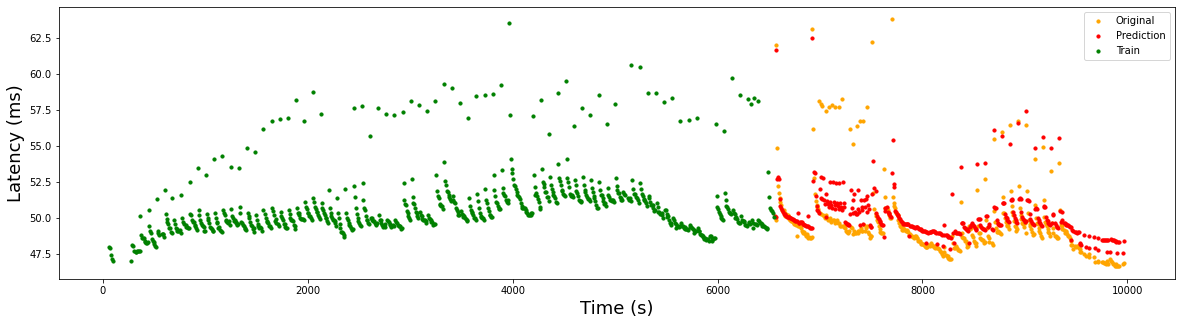
**

**Supplementary Figure 1.** Training latencies, original latencies from MES-I and the predicted latencies resulting from an ARX model with the history of 3 steps and the spike count in the past 8.1 seconds as an exogenous input. The $R^{2}$-score is -0.26.

**
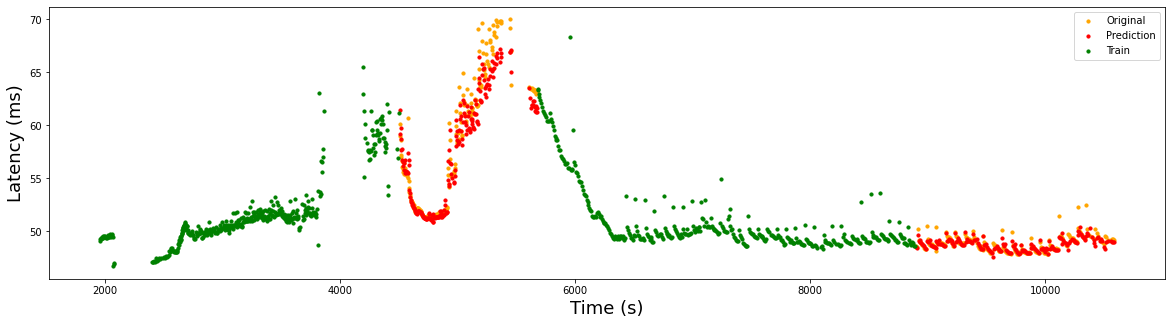
**

**Supplementary Figure 2.** Training latencies, original latencies from MES-II and the predicted latencies resulting from an ARX model with the history of 3 steps and the spike count in the past 8.1 seconds as an exogenous input. The $R^{2}$-score is 0.95.
